# Analytical Solution of Electro-Osmotic Peristalsis of Fractional Jeffreys Fluid in a Micro-Channel

**DOI:** 10.3390/mi8120341

**Published:** 2017-11-23

**Authors:** Xiaoyi Guo, Haitao Qi

**Affiliations:** 1 School of Mathematics and Statistics, Linyi University, Linyi 276000, China; 2 School of Mathematics and Statistics, Shandong University, Weihai 264209, China; htqi@sdu.edu.cn

**Keywords:** electro-osmotic flow, peristalsis, fractional calculus, Jeffreys fluid, Laplace transform

## Abstract

The electro-osmotic peristaltic flow of a viscoelastic fluid through a cylindrical micro-channel is studied in this paper. The fractional Jeffreys constitutive model, including the relaxation time and retardation time, is utilized to describe the viscoelasticity of the fluid. Under the assumptions of long wavelength, low Reynolds number, and Debye-Hückel linearization, the analytical solutions of pressure gradient, stream function and axial velocity are explored in terms of Mittag-Leffler function by Laplace transform method. The corresponding solutions of fractional Maxwell fluid and generalized second grade fluid are also obtained as special cases. The numerical analysis of the results are depicted graphically, and the effects of electro-osmotic parameter, external electric field, fractional parameters and viscoelastic parameters on the peristaltic flow are discussed.

## 1. Introduction

The electro-osmotic transport in micro-channels and nano-channels recently attract much attentions of researchers because of the development of biomedical microelectromechanical systems (Bio-MEMS) and lab-on-a-chip technologies. It has wide applications in biomedicine, biology, and biotechnology. Various experimental, theoretical, and numerical investigations of electro-kinetics have been presented in previous studies. For example, Sadr et al. [1] experimentally studied electro-osmotic flow in rectangular microchannels. Santiago et al. [2] theoretically analyzed the effects of fluid inertia and pressure on the velocity and vorticity field of electro-osmotic flows. The effects of the electrical double layer near the solid/liquid interface on liquid flow through a rectangular microchannel are discussed by Yang et al. [3]. Wang et al. [4] investigated the electro-osmotic flow through a microchannel with a semicircular cross-section and gave analytical series solutions for two basic cases. Jian et al. [5] studied the flow behavior of time periodic electro-osmosis in a cylindrical microannulus. An analytical solution of electro-osmotic flow (EOF) velocity distribution as functions of radial distance, periodic time, and relevant parameters are derived. Finite difference simulation was also applied to the problems of electro-osmosis [6]. Furthermore, taking most biofluids such as blood emerge the viscoelastic feature into consideration, more and more interests are being shown in the electro-osmotic flow of non-Newtonian fluids. Das and Chakraborty [7,8] firstly studied the non-Newtonian effects on electro-osmotic flow. Then power-law fluid, second-grade fluid, Maxwell fluid, and Oldroyd-B fluid were discussed as the model of viscoelastic fluid in electro-osmotic flow by Zhao et al. [9,10,11,12,13].

Additionally, peristaltic flow, which is generated by means of contraction and expansion of the tube and channel wall, also has wide applications in many physiological processes and industries. Peristalsis, or the mechanism of peristalsis, is used to propel the biological fluid from one organ to another—for instance, the transport of blood in vessels and the movement of the chyme in the digestive system. Since Latham [14] and Fung et al. [15], many investigations of peristalsis for Newtonian and non-Newtonian fluids have been carried out theoretically and experimentally [16,17,18,19,20,21]. Hayat et al. [22,23,24,25,26] further researched the effects of the imposed magnetic field and porous medium on the peristaltic flow. Rashidi et al. [27] described entropy generation on magneto-hydrodynamic (MHD) blood flow of nanofluid due to peristaltic waves.

With recent advances in mechatronics, some effective attempts have been made for regarding peristaltic flow with applied electric fields. Bandopadhya et al. [28] investigated the peristaltic motion of an aqueous electrolyte with an externally-applied electric field along a finite channel. They obtained the spatial distribution of pressure and wall shear stress for a continuous wave and single pulse peristaltic wave. Goswami et al. [29] discussed the electrokinetically modulated peristaltic transport of power-law fluids. The fluid is considered to be divided into two regions—a non-Newtonian core region which is surrounded by a thin wall-adhering layer of Newtonian fluid. The effects of fluid viscosities, power-law index and electro-osmosis on pumping characteristics and the trapping of the fluid bolus are studied. Tripathi et al. [30,31] respectively established a mathematical model for electro-osmotic flow of non-Newtonian fluids in a micro-channel and the Newtonian viscous model of electro-osmotic modulated capillary peristalsis in a finite length vessel. In the two articles, the influence of electro-osmotic parameter and maximum electro-osmotic velocity on axial velocity, volumetric flow rate, pressure gradient, local wall shear stress, and stream function distributions were evaluated in detail.

As we know, the fractional model is more flexible to describe the viscoelastic property of the non-Newtonian fluids in physics, biology and medical engineering, because a very good fit of experimental data can be achieved when the constitutive equation with fractional derivative is used [32]. The fractional viscoelastic fluid model has attracted wide attention in modern mechanics [33,34,35]. Recently, peristaltic transport of fractional viscoelastic fluid in different system began to play an important role through the work [36,37,38,39]. Additionally, the electro-osmotic flow of fractional viscoelastic fluid was discussed in [40,41,42,43]. To the best of the authors’ knowledge, very few articles have discussed fractional models of electro-osmotic peristaltic flow.

Based on the promising results achieved in the above literature review, this paper attempts to study the fractional Jeffreys model of electro-osmotic peristaltic flow in an infinite micro-channel, with an aim to analyze the effects of electro-osmosis and fractional parameter on peristaltic flow of fractional viscoelastic fluid. The fractional calculus is taken into account in the Jeffrey’s version of the Oldroyd constitutive model. This fluid model, which includes time derivatives of the stress tensor and a derivative of the rate of strain tensor, shows relaxation as well as retardation behavior [22,44]. Using integrated method and Laplace transform, we investigate the solutions of velocity distribution, pressure gradient and stream function under assumptions of the long wavelength, low Reynolds number, and Debye-Hückel linearization. The paper is structured as follows: Section 1 serves as an introduction; Section 2 deduces the basic equations of the fluid and presents the initial and boundary value problem for the flow; Section 3 discusses the analytical solution to the problem; Section 4 discusses the special cases and the numerical results; and Section 5 draws a conclusion for the whole paper.

## 2. Basic Equations

According to the incompressible fluid of the classical Jeffreys model [22,44], the constitutive relationship of the fractional Jeffreys fluid is given by [40]:

(1) $$\left(1+\lambda_{1}^{\alpha }D_{t}^{\alpha }\right)\tau \begin{array}{cc} =\mu \left(1+\lambda_{2}^{\beta }D_{t}^{\beta }\right)\dot{\gamma }, & \;0\leq \alpha \leq \beta \leq 1 \end{array}$$

where *τ* is the shear stress tensor, $\dot{\gamma }$ is the rate of strain tensor, *μ* is the viscosity of the fluid, *λ*_1_ and *λ*_2_ are constant relaxation and retardation times, $D_{t}^{\alpha }$ and $D_{t}^{\beta }$ are the fractional calculus of order *α* and *β* with respect to *t*, respectively, and may be defined as [45]:

(2) $$D_{t}^{p}f\left(t\right)=\frac{1}{\Gamma \left(1-p\right)}\frac{d}{dt}{\int_{0}^{t}\left(t-\tau \right)}^{-p}f\left(\tau \right)d\tau ,\begin{array}{cc} & 0\leq p \end{array}\leq 1.$$

This model includes the ordinary Jeffreys fluid as a special case for *α* = *β* = 1, in which *λ*_1_ and *λ*_2_ are relaxation and retardation time. This model also can be simplified to be the generalized second-grade fluid when *α* = 0, *λ*_1_ → 0 and to be the fractional Maxwell fluid when *β* = 0, *λ*_2_ → 0.

**Figure 1:**
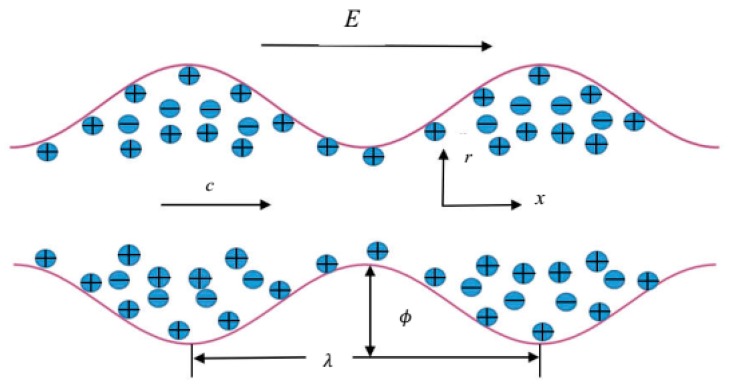
Geometry of the problem.

We consider the unsteady electro-osmotic peristaltic transport of a viscoelastic fluid through a cylindrical channel with an externally-applied electric field along the axial direction (Figure 1). The geometry of the channel wall is mathematically described as follows:

(3) $$h=a-\phi \cos^{2}\frac{\pi }{\lambda }\left(x-ct\right)$$

where *a* is the radius of the channel, ϕ, *λ*, *c*, and *t* are the amplitude, wavelength, wave velocity, and time, respectively. It is assumed that the channel is filled with an ionic solution, such as blood, which may be manipulated by the external electric field. According to the theory of electrostatics, the Poisson-Boltzmann equation to describe the electric potential distribution for a symmetric binary electrolyte solution is given as [31]:

(4) $$\nabla^{2}\Phi =-\frac{\rho_{e}}{\varepsilon }$$

in which $\rho_{e}$ is the net charge density, ε is the permittivity. When there is no axial gradient of the ionic concentration within the micro-channel and the Debye-Hückel linearization approximation is employed [31], we have:

(5) $$\rho_{e}=-\frac{2n_{0}z^{2}e^{2}\Phi }{K_{B}T}$$

where $n_{0}$ is the ion density of the bulk, z is the valence of ions, *e* is electronic charge, *K_B_* is the Boltzmann constant, *T* is the average temperature of the electronic solution.

The governing equations describing the considering flow of the incompressible fluid are:

(6) divV=0

(7) $$\rho \frac{dV}{dt}=-\nabla p+\mathrm{div}\tau -\rho_{e}E$$

where d/dt is the material time derivative, **V** is the velocity vector, ρ is the density, *p* is the pressure, **E** is the applied external electric field. In the axisymmetric cylindrical coordinate system (*x*, *r*), in which variable *r* is radial coordinate and *x*-axis along the center line of the channel, we have:

(8) $$\frac{\partial u}{\partial x}+\frac{1}{r}\frac{\partial \left(rv\right)}{\partial r}=0,$$

(9) $$\begin{array}{l} \rho \left(1+\lambda_{1}^{\alpha }D_{t}^{\alpha }\right)\left(\frac{\partial u}{\partial t}+u\frac{\partial u}{\partial x}+v\frac{\partial u}{\partial r}\right)=-\left(1+\lambda_{1}^{\alpha }D_{t}^{\alpha }\right)\frac{\partial p}{\partial x}+\\ \mu \left(1+\lambda_{2}^{\beta }D_{t}^{\beta }\right)\left(\frac{\partial^{2}u}{\partial x^{2}}+\frac{1}{r}\frac{\partial }{\partial r}\left(r\frac{\partial u}{\partial r}\right)\right)+\left(1+\lambda_{1}^{\alpha }D_{t}^{\alpha }\right)\rho_{e}E_{x} \end{array}$$

(10) $$\begin{array}{l} \rho \left(1+\lambda_{1}^{\alpha }D_{t}^{\alpha }\right)\left(\frac{\partial v}{\partial t}+u\frac{\partial v}{\partial x}+v\frac{\partial v}{\partial r}\right)=-\left(1+\lambda_{1}^{\alpha }D_{t}^{\alpha }\right)\frac{\partial p}{\partial r}+\\ \mu \left(1+\lambda_{2}^{\beta }D_{t}^{\beta }\right)\left(\frac{\partial^{2}v}{\partial x^{2}}+\frac{\partial }{\partial r}\left(\frac{1}{r}\frac{\partial \left(rv\right)}{\partial r}\right)\right) \end{array}$$

where, *u* and *v* denote the axial velocity and radial velocity, respectively.

We introduce dimensionless variables and parameters as follow:

x^=xλ,r^=ra,t^=ctλ,u^=uc,v^=vcδ,λ^1=cλ1λ,λ^2=cλ2λ,p^=pa02μcλ,ϕ^=ϕa,h^=ha=1−ϕ^cos2π(x^−t^),Φ^=Φζ,δ=aλ,Re=ρcaμ

in which *δ*(≪1), Re, and ζ are the wave number, Reynolds number, and zeta potential of the channel wall, respectively.

Then under the approximations of the long wavelength and low Reynolds number, we obtain the dimensionless equations (for simplicity, the dimensionless mark “^” will be neglected from here on) from Equations (4), (5), and (8)–(10):

(11) $$\frac{1}{r}\frac{\partial }{\partial r}\left(r\left(\frac{\partial \Phi }{\partial r}\right)\right)=m^{2}\Phi$$

(12) $$\frac{\partial u}{\partial x}+\frac{1}{r}\frac{\partial \left(rv\right)}{\partial r}=0$$

(13) $$\left(1+\lambda_{2}^{\beta }D_{t}^{\beta }\right)\frac{1}{r}\frac{\partial }{\partial r}\left(r\frac{\partial u}{\partial r}\right)=\left(1+\lambda_{1}^{\alpha }D_{t}^{\alpha }\right)\left(\frac{\partial p}{\partial x}-m^{2}\Phi U_{\mathrm{HS}}\right)$$

(14) $$\frac{\partial p}{\partial r}=0$$

where *m* = *ae*$z\sqrt{2n_{0}/\varepsilon K_{B}T}$ = $a/\lambda_{d}$ is called the Debye-Hückel parameter, *λ_d_* is the Debye length or characteristic thickness of the electrical double layer (EDL), and $U_{\mathrm{HS}}$ = −$E_{x}\varepsilon \zeta /\mu c$ is the Helmholtz-Smoluchowski velocity.

The corresponding boundary conditions are:

(15) $$\frac{\partial \Phi }{\partial r}=0,\;\mathrm{as}\;r=0;\;\Phi =0,\;\mathrm{as}\;r=h;$$

(16) $$\frac{\partial u}{\partial r}=0,\;\mathrm{as}\;r=0;\;u=0,\;\mathrm{as}\;r=h.$$

## 3. Solution of the Problem

Solving Equation (11) with Equation (15), we obtain the potential distribution:

(17) $$\Phi =\frac{I_{0}\left(mr\right)}{I_{0}\left(mh\right)}$$

where *I*_0_ (·) is the zero-order modified Bessel function of the first kind.

By integrating Equation (13) with respect to *r* and considering Equation (16), the solution for the axial velocity is obtained as:

(18) $$u={\left(1+\lambda_{2}^{\beta }D_{t}^{\beta }\right)}^{-1}\left(1+\lambda_{1}^{\alpha }D_{t}^{\alpha }\right)\left[\frac{1}{4}\frac{\partial p}{\partial x}\left(r^{2}-h^{2}\right)-U_{\mathrm{HS}}\left(\frac{I_{0}\left(mr\right)}{I_{0}\left(mh\right)}-1\right)\right]$$

The volumetric flow rate in the fixed frame is defined as:

(19) $$Q=\int_{0}^{h}2rudr={\left(1+\lambda_{2}^{\beta }D_{t}^{\beta }\right)}^{-1}\left(1+\lambda_{1}^{\alpha }D_{t}^{\alpha }\right)\left[-\frac{h^{4}}{8}\frac{\partial p}{\partial x}+U_{\mathrm{HS}}\left(h^{2}-\frac{2hI_{1}\left(mh\right)}{mI_{0}\left(mh\right)}\right)\right]$$

The relationships of the wave frame (*X*, *R*), (*U*, *V*) moving with velocity *c* and the fixed frame, (*x*, *r*), (*u*, *v*) in dimensionless form, are given by:

(20) X=x−t,R=r,U=u−1,V=v

Thus, the volumetric flow rate in the wave frame is:

(21) $$q=\int_{0}^{h}2RUdR=\int_{0}^{h}2r\left(u-1\right)dr=Q-h^{2}$$

The average of the volumetric flow rate along one time period gives:

(22) $$\bar{Q}=\int_{0}^{1}Qdt=\int_{0}^{1}\left(q+h^{2}\right)dt=q+1-\phi +\frac{3}{8}\phi^{2}$$

From Equations (19)–(22), we can deduce:

(23) $$\frac{\partial p}{\partial X}=\frac{8}{h^{4}}U_{\mathrm{HS}}\left(h^{2}-\frac{2hI_{1}\left(mh\right)}{mI_{0}\left(mh\right)}\right)-{\left(1+\lambda_{1}^{\alpha }D_{t}^{\alpha }\right)}^{-1}\left(1+\lambda_{2}^{\beta }D_{t}^{\beta }\right)\frac{8}{h^{4}}\left(\bar{Q}+h^{2}-1+\phi -\frac{3}{8}\phi^{2}\right)$$

Then, from Equation (18), we have:

(24) $$\begin{array}{l} U={\left(1+\lambda_{2}^{\beta }D_{t}^{\beta }\right)}^{-1}\left(1+\lambda_{1}^{\alpha }D_{t}^{\alpha }\right)\left[\frac{2\left(r^{2}-h^{2}\right)}{h^{4}}U_{\mathrm{HS}}\left(h^{2}-\frac{2hI_{1}\left(mh\right)}{mI_{0}\left(mh\right)}\right)-U_{\mathrm{HS}}\left(\frac{I_{0}\left(mr\right)}{I_{0}\left(mh\right)}-1\right)\right]-\\ \frac{2\left(r^{2}-h^{2}\right)}{h^{4}}\left(\bar{Q}+h^{2}-1+\phi -\frac{3}{8}\phi^{2}\right)-1 \end{array}$$

Let *f*(*X*, *t*) = ∂p/∂X. Then, in order to give the analytical solution of the pressure gradient in the wave frame, we introduce the Laplace transform:

(25) $$\tilde{f}\left(X,s\right)=L\left[f\left(X,t\right),s\right]=\int_{0}^{\infty }f\left(X,t\right)e^{-st}dt$$

Considering the initial condition *f*(*X*, 0) = 0 and the Laplace transform formula of the fractional derivative [45], the transform of Equation (23) is given as:

(26) $$\tilde{f}\left(X,s\right)=\frac{8}{sh^{4}}U_{\mathrm{HS}}\left(h^{2}-\frac{2hI_{1}\left(mh\right)}{mI_{0}\left(mh\right)}\right)-\frac{1+\lambda_{2}^{\beta }s^{\beta }}{s\left(1+\lambda_{1}^{\alpha }s^{\alpha }\right)}\frac{8}{h^{4}}\left(\bar{Q}+h^{2}-1+\phi -\frac{3}{8}\phi^{2}\right)$$

Finally, applying the inverse Laplace transform we obtain:

(27) $$\begin{array}{c} \frac{\partial p}{\partial X}=\frac{8}{h^{4}}U_{\mathrm{HS}}\left(h^{2}-\frac{2hI_{1}\left(mh\right)}{mI_{0}\left(mh\right)}\right)-L^{-1}\left[\frac{1+\lambda_{2}^{\beta }s^{\beta }}{s\left(1+\lambda_{1}^{\alpha }s^{\alpha }\right)}\right]\frac{8}{h^{4}}\left(\bar{Q}+h^{2}-1+\phi -\frac{3}{8}\phi^{2}\right)\\ =\frac{8}{h^{4}}U_{\mathrm{HS}}\left(h^{2}-\frac{2hI_{1}\left(mh\right)}{mI_{0}\left(mh\right)}\right)-\left[\frac{t^{\alpha }}{\lambda_{1}^{\alpha }}E_{\alpha ,\alpha +1}\left(-\frac{t^{\alpha }}{\lambda_{1}^{\alpha }}\right)+\frac{t^{\alpha -\beta }\lambda_{2}^{\beta }}{\lambda_{1}^{\alpha }}E_{\alpha ,\alpha -\beta +1}\left(-\frac{t^{\alpha }}{\lambda_{1}^{\alpha }}\right)\right]\\ \times \frac{8}{h^{4}}\left(\bar{Q}+h^{2}-1+\phi -\frac{3}{8}\phi^{2}\right) \end{array}$$

where $E_{p,\;q}\left(\cdot \right)$ is the Mittag-Leffler function [45], defined by $E_{p,\;q}\left(z\right)=\overset{\infty }{\underset{k=0}{\sum }}z^{k}/\Gamma \left(pk+q\right)$.

According to the definition of fractional operator, the analytical solution of axial velocity in wave frame is also obtained:

(28) $$\begin{array}{l} U=\left[\frac{2\left(r^{2}-h^{2}\right)}{h^{4}}U_{\mathrm{HS}}\left(h^{2}-\frac{2hI_{1}\left(mh\right)}{mI_{0}\left(mh\right)}\right)-U_{\mathrm{HS}}\left(\frac{I_{0}\left(mr\right)}{I_{0}\left(mh\right)}-1\right)\right]\times \\ {\left(1+\frac{\lambda_{2}^{\beta }t^{\beta }}{\Gamma \left(1-\beta \right)}\right)}^{-1}\left(1+\frac{\lambda_{1}^{\alpha }t^{\alpha }}{\Gamma \left(1-\alpha \right)}\right)-\frac{2\left(r^{2}-h^{2}\right)}{h^{4}}\left(\bar{Q}+h^{2}-1+\phi -\frac{3}{8}\phi^{2}\right)-1 \end{array}$$

Since in wave frame the velocity U = $\frac{1}{R}\frac{\partial \psi }{\partial R}$, in which *ψ* is the stream function, we can give:

(29) $$\begin{array}{l} \psi =\left[\frac{R^{4}-R^{2}h^{2}}{2h^{4}}U_{\mathrm{HS}}\left(h^{2}-\frac{2hI_{1}\left(mh\right)}{mI_{0}\left(mh\right)}\right)-U_{\mathrm{HS}}\left(\frac{RI_{1}\left(mR\right)}{mI_{0}\left(mh\right)}-\frac{R^{2}}{2}\right)\right]\times \\ {\left(1+\frac{\lambda_{2}^{\beta }t^{\beta }}{\Gamma \left(1-\beta \right)}\right)}^{-1}\left(1+\frac{\lambda_{1}^{\alpha }t^{\alpha }}{\Gamma \left(1-\alpha \right)}\right)-\frac{R^{4}-R^{2}h^{2}}{2h^{4}}\left(\bar{Q}+h^{2}-1+\phi -\frac{3}{8}\phi^{2}\right)-\frac{R^{2}}{2} \end{array}$$

The dimensionless pressure rise and friction can be obtained as follows:

(30) $$\Delta p=\int_{0}^{1}\frac{\partial p}{\partial X}dX$$

(31) $$F=\int_{0}^{1}-h^{2}\frac{\partial p}{\partial X}dX$$

## 4. Discussion and Numerical Results

In the special case, if *α* = 0, *λ*_1_ → 0, corresponding to the electro-osmotic peristaltic flow of generalized second grade fluid (GSF), the pressure gradient reduces to:

(32) $$\frac{\partial p}{\partial X}=\frac{8}{h^{4}}U_{\mathrm{HS}}\left(h^{2}-\frac{2hI_{1}\left(mh\right)}{mI_{0}\left(mh\right)}\right)-\frac{8}{h^{4}}\left(1+\frac{\lambda_{2}^{\beta }t^{\beta }}{\Gamma \left(1-\beta \right)}\right)\left(\bar{Q}+h^{2}-1+\phi -\frac{3}{8}\phi^{2}\right)$$

When *β* = 0 and *λ*_2_ → 0, Equation (27) can be simplified to:

(33) $$\frac{\partial p}{\partial X}=\frac{8}{h^{4}}U_{\mathrm{HS}}\left(h^{2}-\frac{2hI_{1}\left(mh\right)}{mI_{0}\left(mh\right)}\right)-\frac{8t^{\alpha }}{h^{4}\lambda_{1}^{\alpha }}E_{\alpha ,\alpha +1}\left(-\frac{t^{\alpha }}{\lambda_{1}^{\alpha }}\right)\left(\bar{Q}+h^{2}-1+\phi -\frac{3}{8}\phi^{2}\right)$$

which is the pressure gradient for fractional Maxwell fluid (FMF).

If *α* =0, *λ*_1_ → 0, *β* = 0 and *λ*_2_ → 0, we obtain the solution of the electro-osmotic peristaltic flow for Newtonian fluid from (27) as follows:

(34) $$\frac{\partial p}{\partial X}=\frac{8}{h^{4}}U_{\mathrm{HS}}\left(h^{2}-\frac{2hI_{1}\left(mh\right)}{mI_{0}\left(mh\right)}\right)-\frac{8}{h^{4}}\left(\bar{Q}+h^{2}-1+\phi -\frac{3}{8}\phi^{2}\right)$$

which is the same solution given by Tripathi et al. [31]. 

**Figure 2:**
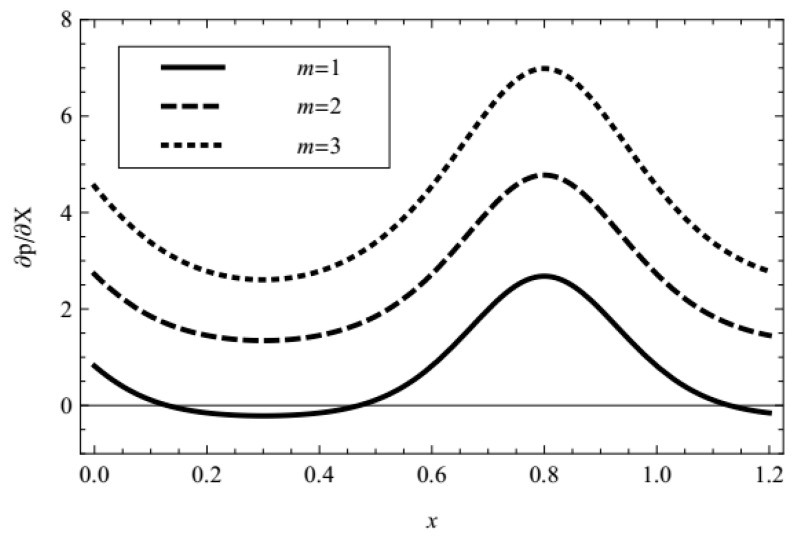
Profiles of the pressure gradient for various values of *m* with fixed *α* = 0.4, *β* = 0.6, *λ*_1_ = 1, *λ*_2_ = 1, $U_{\mathrm{HS}}$ = 1, ϕ = 0.3, $\bar{Q}$ = 0.1, *t* = 0.8.

**Figure 3:**
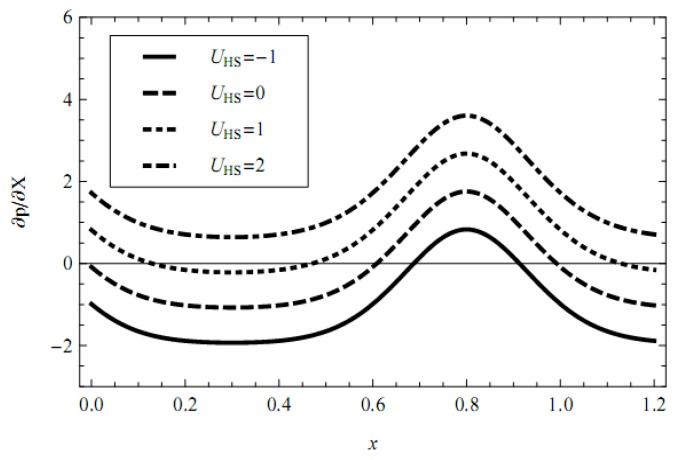
Profiles of the pressure gradient for various values of $U_{\mathrm{HS}}$ with fixed *α* = 0.4, *β* = 0.6, *λ*_1_ = 1, *λ*_2_ = 1, *m* = 1, ϕ = 0.3, $\bar{Q}$ = 0.1, *t* = 0.8.

**Figure 4:**
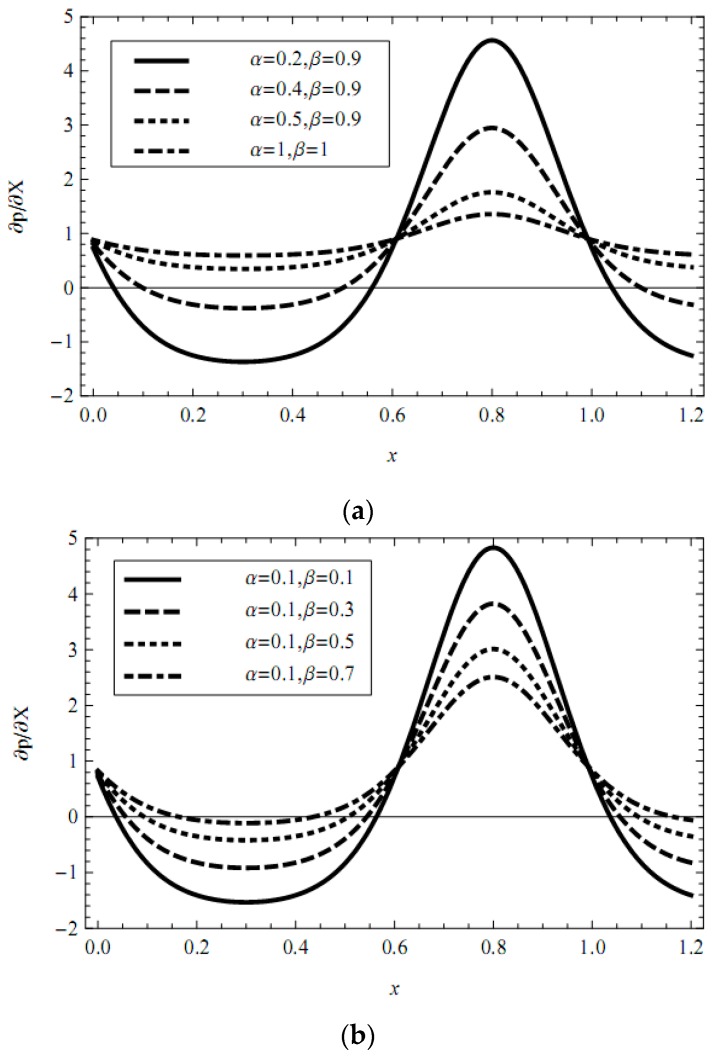
Profiles of the pressure gradient for various values of fractional operator parameter (**a**) *α* and (**b**) *β* with fixed *λ*_1_ = 1, *λ*_2_ = 1, *m* = 1, ϕ = 0.3, $U_{\mathrm{HS}}$ = 1, $\bar{Q}$ = 0.1, *t* = 0.8.

**Figure 5:**
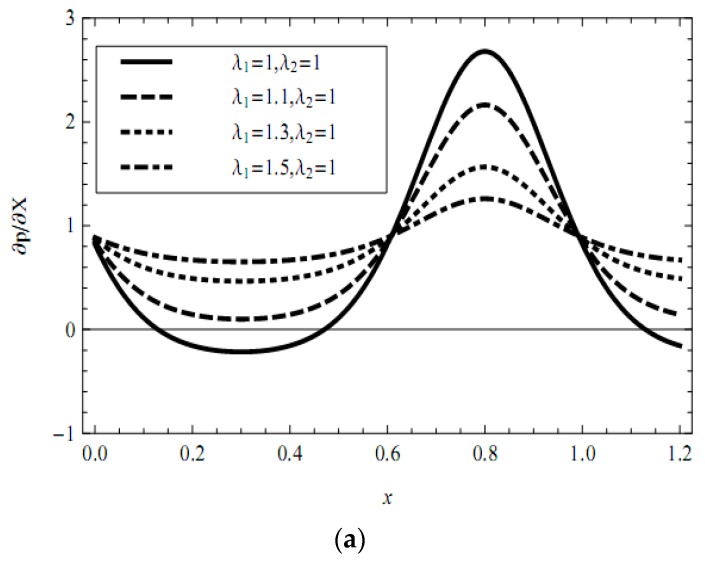

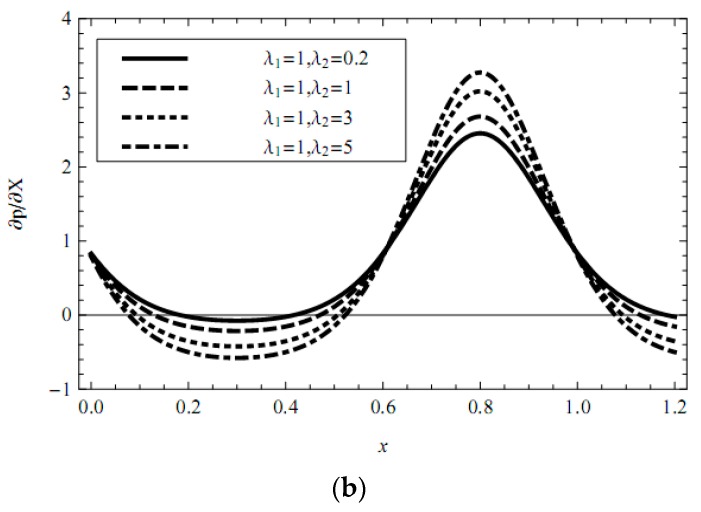
Profiles of the pressure gradient for various values of material parameter (**a**) *λ*_1_ and (**b**) *λ*_2_ with fixed *α* = 0.4, *β* = 0.6, *m* = 1, ϕ = 0.3, $U_{\mathrm{HS}}$ = 1, $\bar{Q}$ = 0.1, *t* = 0.8.

**Figure 6:**
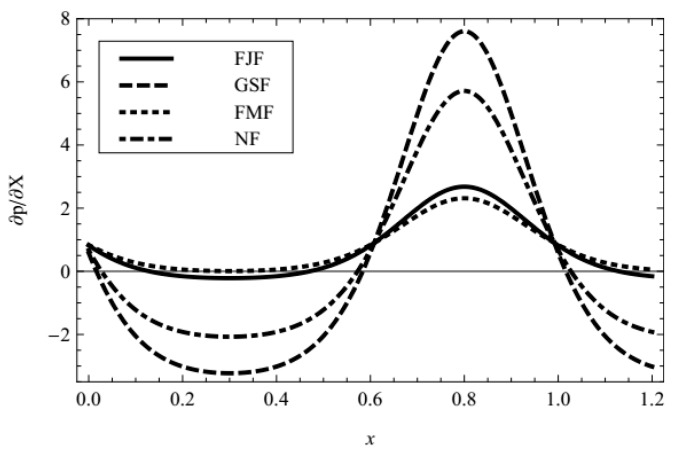
Profiles of the pressure gradient for fractional Jeffreys fluid (FJF) (*α* = 0.4, *β* = 0.6, *λ*_1_ = 1, *λ*_2_ = 1), generalized second fluid (GSF) (*α* = 0, *β* = 0.6, *λ*_1_ → 0, *λ*_2_ = 1), fractional Maxwell fluid (FMF) (*α* = 0.4, *β* = 0, *λ*_1_ = 1, *λ*_2_ → 0), and Newtonian fluid (NF), with fixed *m* = 1, ϕ = 0.3, $U_{\mathrm{HS}}$ = 1, $\bar{Q}$ = 0.1, *t* = 0.8.

In addition, the influences of pertinent parameters on the flow motion are discussed through graphical illustrations. Firstly, we consider the effects of characteristic thickness of EDL, external electric field, fractional parameters, and viscoelastic parameters on the pressure gradient. Figure 2, Figure 3, Figure 4, Figure 5 and Figure 6 present the axial pressure gradient profiles, i.e., the pressure gradient vs. the axial coordinate along the center line of the channel in one period with fixed time and flow rate. Evidently the pressure gradient profiles are uniform and exhibits periodicity due to the nature of the peristaltic flow, i.e., it is at a minimum at fully-relaxed wall locations and exhibits maximum values at fully-contracted wall locations. In Figure 2, Figure 3, Figure 4, Figure 5 and Figure 6 we find also that, because of the contraction and relaxation of the walls of the channel, there always exists a negative pressure gradient which causes the forward propagation of the trapped bolus.

Figure 2 depicts the pressure gradient for increasing Debye-Hückel parameter (which is inversely proportional to Debye length or characteristic thickness of EDL). It is noticed that the pressure gradient is elevated with the increasing Debye-Hückel parameter, i.e., with decreasing characteristic thickness of EDL, and the decreasing characteristic thickness of EDL can reduce the negative pressure gradient.

The effect of the Helmholtz-Smoluchowski velocity (which is proportional to external electric field) on the pressure gradient is shown in Figure 3. It is observed that, with an increase in the Helmholtz-Smoluchowski velocity, i.e., with an increase in the external electric field there is a consistent enhancement in the pressure gradient, and the increasing external electric field strength can restrain the negative pressure gradient.

In Figure 4a,b, we show the effects of the fractional parameters (*α*, *β*) on the pressure gradient. Figure 4a depicts the pressure gradient profiles as *α* is increased from 0.2 through 0.4 to 0.5 with *β* fixed at 0.9. The increase in parameter *α* is observed to reduce the pressure gradient at contracted wall locations, and to enhance the pressure gradient at relaxed wall locations, inversely. Figure 4a also depicts the pressure gradient for classical Jeffreys fluid (*α* = *β* = 1). We find that the changes in pressure gradient of the fractional Jeffreys fluid are more significant than that of classical Jeffreys fluid. Figure 4b shows that the pressure gradient decreases with increasing *β* at contracted wall locations and inversely at relaxed wall locations.

Figure 5a,b illustrates the effects of viscoelastic parameters (relaxation time and retardation time) on the pressure gradient distribution. In Figure 5a, the maximum value of pressure gradient decreases with increasing relaxation time, while the minimum value increases. Inversely, in Figure 5b, the maximum value of pressure gradient enhances with greater value of retardation time, while the minimum reduces. It is concluded that the elasticity of the viscoelastic fluid suppresses the negative pressure gradient because, in general, for the viscoelastic model with greater relaxation time, the fluid is more elastic.

For comparison, the pressure gradient of fractional Jeffreys fluid (FJF), generalized second fluid (GSF), fractional Maxwell fluid (FMF), and Newtonian fluid (NF) are plotted in Figure 6. It is observed that the amplitude of the pressure gradient of fractional Jeffreys fluid falls in between that of generalized second fluid and fractional Maxwell fluid in the same situation.

**Figure 7:**
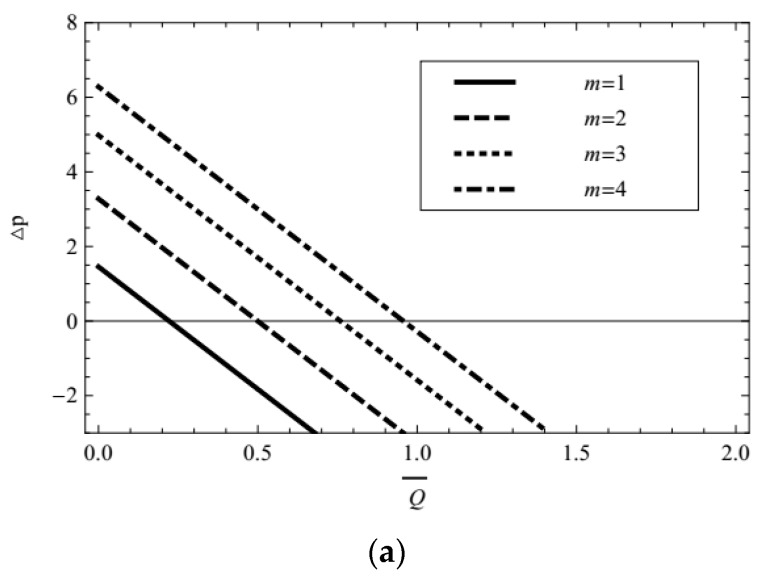

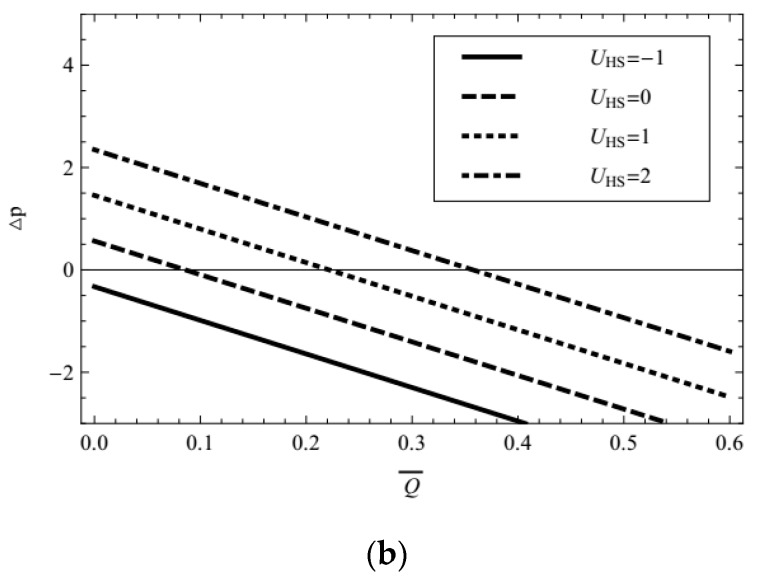
Profiles of pressure rise vs. time averaged volumetric flow rate for (**a**) $U_{\mathrm{HS}}$ = 1 (**b**) *m* = 1, with fixed *α* = 0.4, *β* = 0.6, *λ*_1_ = 1, *λ*_2_ = 1, ϕ = 0.3, *t* = 0.8.

The profile of pressure rise vs. the time averaged volumetric flow rate for different values of Debye-Hückel parameter and Helmholtz-Smoluchowski velocity are given in Figure 7a,b. Obviously the pressure rise is the linear function of the flow rate, and decreases with the greater flow rate. Figure 7a shows that increasing Debye-Hückel parameter, i.e., decreasing characteristic thickness of EDL elevates the pressure rise remarkably. As the Helmholtz-Smoluchowski velocity (i.e., external electric field) increases, Figure 7b illustrates that there is also a consistent elevation in the pressure rise. Especially when *U*_HS_ = −1, the pressure is always negative for all flow rates.

**Figure 8:**
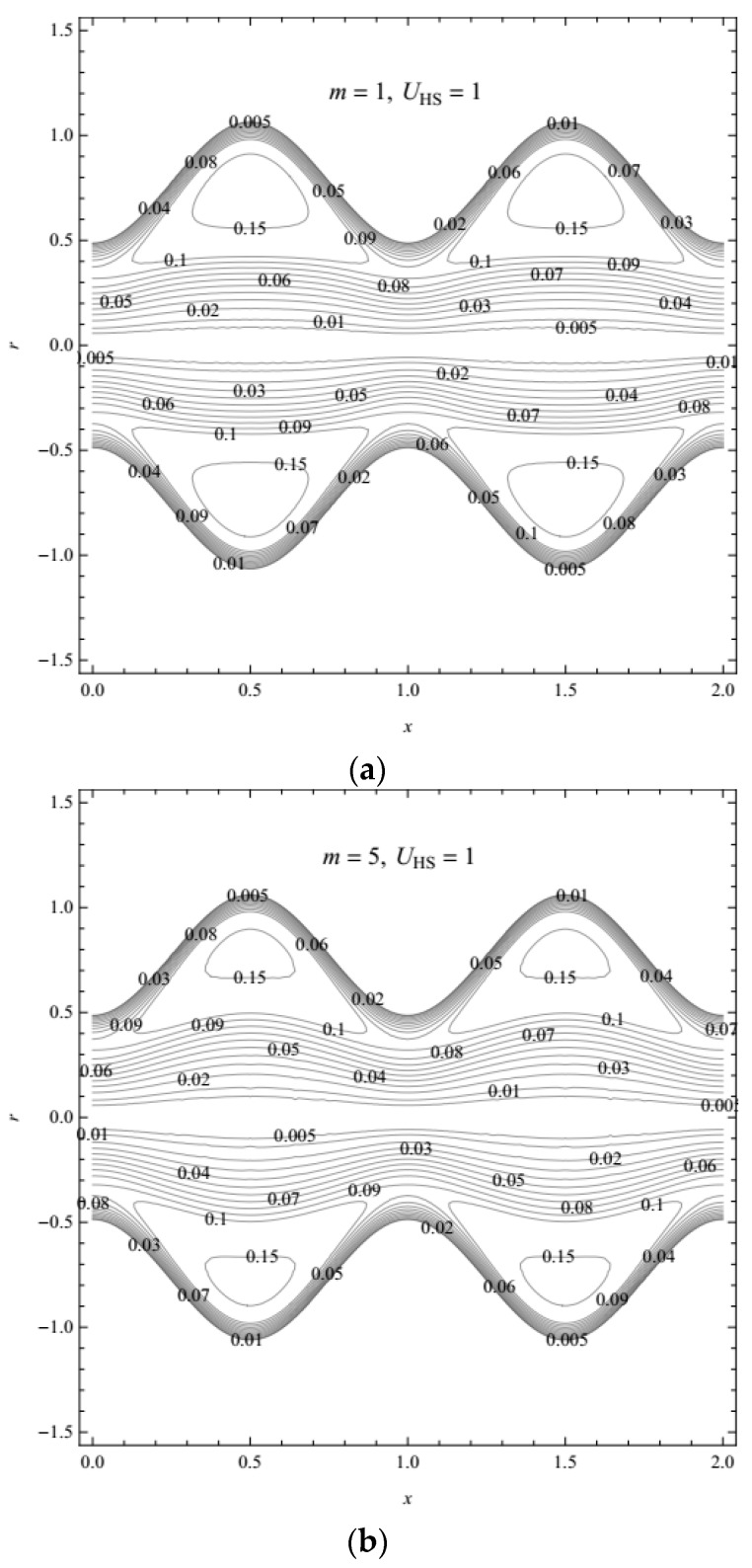

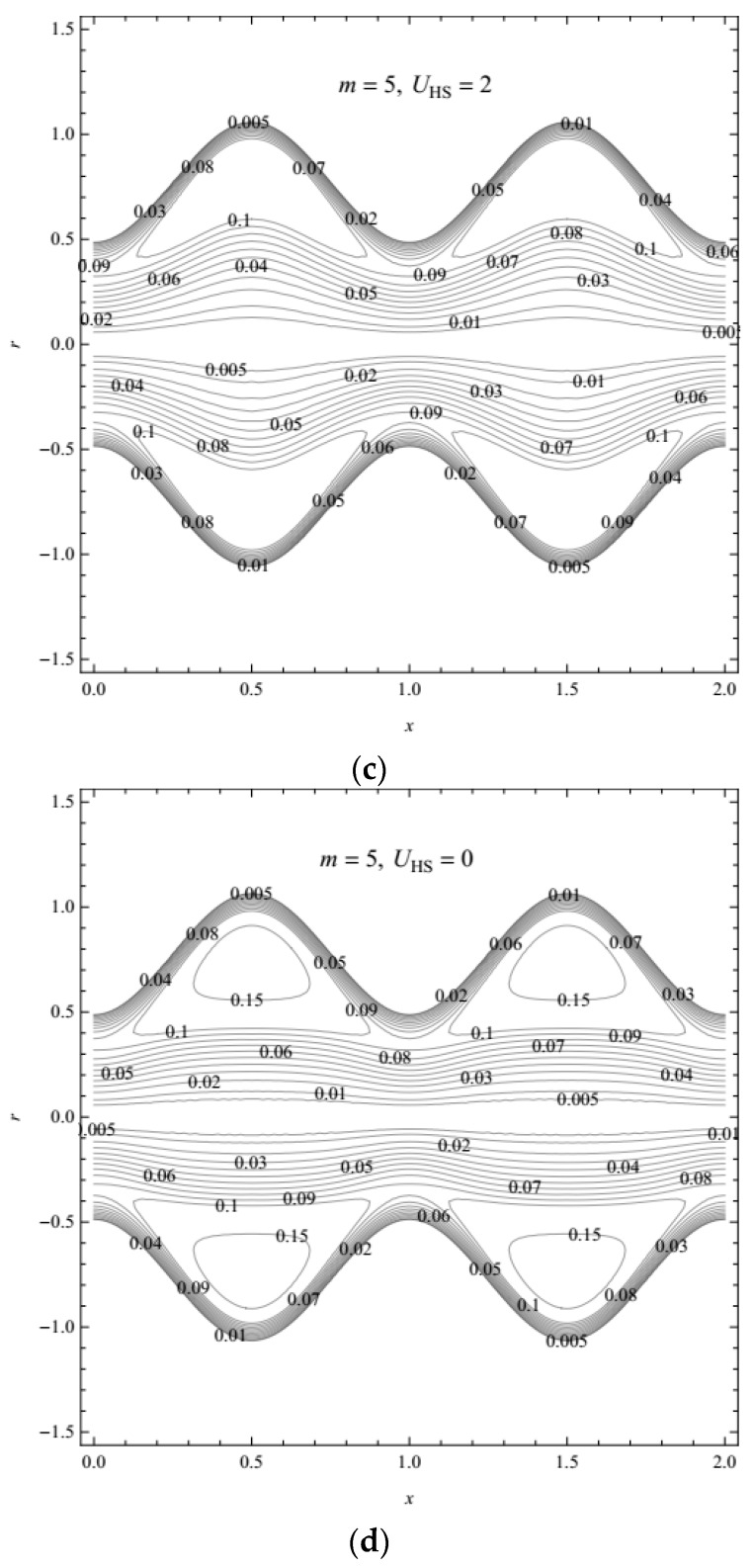
Profiles of the stream function for different values of *m* and $U_{\mathrm{HS}}$: (**a**) *m* = 1, $U_{\mathrm{HS}}$ = 1; (**b**) *m* = 5, $U_{\mathrm{HS}}$ = 1; (**c**) *m* = 5, $U_{\mathrm{HS}}$ = 2; (**d**) *m* = 5, $U_{\mathrm{HS}}$ = 0 with fixed *α* = 0.4, *β* = 0.6, *λ*_1_ = 1, *λ*_2_ = 1, ϕ = 0.6, *t* = 0.8.

Furthermore, trapping, as an important phenomenon of peristaltic transport, is considered. In Figure 8a–d, we give the stream function profiles (radial coordinates versus axial coordinates) for different values of the Debye-Hückel parameter and Helmholtz-Smoluchowski velocity (with and without an external electric field). In Figure 8a,b, it is evident that with the increasing Debye-Hückel parameter, i.e., the decreasing characteristic thickness of EDL, the range of trapped boluses is reduced. In Figure 8b–d, it is observed that when the external electric field is strong enough, the number of trapped boluses significantly reduces with a larger value of the Helmholtz-Smoluchowski velocity (which is proportional to the external electric field).

## 5. Conclusions

In this paper, a mathematic model of the electro-osmotic peristaltic flow for fractional Jeffreys fluid through a cylindrical micro-channel is established. Considering the assumptions of long wavelength, low Reynolds number and Debye-Hückel linearization, the analytical solution of pressure gradient, velocity, and streamline function are presented by using integral transform method of fractional operater. The solutions of the corresponding flow for fractional Maxwell fluid, generalized second fluid, and Newtonian fluid are discussed as the special cases. The visualization solutions via Mathematica software have been shown to evaluate the effects of the electro-osmotic parameter (i.e., Debye-Hückel parameter), external electric field, fractional parameters (*α*, *β*), and viscoelastic parameters on the peristaltic flow and trapping phenomena. The present computations have shown that:

- The axial pressure gradient is elevated with the increasing Debye-Hückel parameter (i.e., decreasing characteristic thickness of EDL) and the increasing external electric field in all ranges of the axial coordinate. In contracted wall locations, the pressure gradient decreases with the increasing fractional parameter (*α*, *β*) and relaxation time, and also with decreasing retardation time. In relaxed wall locations, the converse response is observed and the negative pressure gradient is generated in some cases.
- The pressure rise increases with the increasing Debye-Hückel parameter (i.e., decreasing characteristic thickness of EDL) and the increasing external electric field in all ranges of time-averaged volumetric flow rate. When the pressure rise is fixed, the flow rate is increased with a decrease in the characteristic thickness of EDL and an increase in the external electric field. The influence of the characteristic thickness of EDL on the pressure rise is more remarkable than that of the external electric field.
- The trapped boluses are suppressed with the increasing Debye-Hückel parameter, i.e., a decreasing characteristic thickness of EDL and with a larger value of the Helmholtz-Smoluchowski velocity (which is proportional to the external electric field).
